# Rapid Identification of the Foodborne Pathogen *Trichinella* spp. by Matrix-Assisted Laser Desorption/Ionization Mass Spectrometry

**DOI:** 10.1371/journal.pone.0152062

**Published:** 2016-03-21

**Authors:** Anne Mayer-Scholl, Jayaseelan Murugaiyan, Jennifer Neumann, Peter Bahn, Sabine Reckinger, Karsten Nöckler

**Affiliations:** 1 Department of Biological Safety, Federal Institute for Risk Assessment, Berlin, Germany; 2 Centre for Infectious Medicine, Institute of Animal Hygiene and Environmental Health, Veterinary Faculty, Free University Berlin, Berlin, Germany; Instituto de Diagnostico y Referencia Epidemiologicos, MEXICO

## Abstract

Human trichinellosis occurs through consumption of raw or inadequately processed meat or meat products containing larvae of the parasitic nematodes of the genus *Trichinella*. Currently, nine species and three genotypes are recognized, of which *T*. *spiralis*, *T*. *britovi* and *T*. *pseudospiralis* have the highest public health relevance. To date, the differentiation of the larvae to the species and genotype level is based primarily on molecular methods, which can be relatively time consuming and labor intensive. Due to its rapidness and ease of use a matrix assisted laser desorption / ionization time of flight mass spectrometry (MALDI-TOF MS) reference spectra database using *Trichinella* strains of all known species and genotypes was created. A formicacid/acetonitrile protein extraction was carried out after pooling 10 larvae of each *Trichinella* species and genotype. Each sample was spotted 9 times using α-cyano 4-hydoxy cinnamic acid matrix and a MicroFlex LT mass spectrometer was used to acquire 3 spectra (m/z 2000 to 20000 Da) from each spot resulting in 27 spectra/species or genotype. Following the spectra quality assessment, Biotyper software was used to create a main spectra library (MSP) representing nine species and three genotypes of *Trichinella*. The evaluation of the spectra generated by MALDI-TOF MS revealed a classification which was comparable to the results obtained by molecular methods. Also, each *Trichinella* species utilized in this study was distinct and distinguishable with a high confidence level. Further, different conservation methods such as freezing and conservation in alcohol and the host species origin of the isolated larvae did not have a significant influence on the generated spectra. Therefore, the described MALDI-TOF MS can successfully be implemented for both genus and species level identification and represents a major step forward in the use of this technique in foodborne parasitology.

## Introduction

Nematodes of the genus *Trichinella* are one of the ten foodborne parasites with the greatest global impact [1]. Human trichinellosis has been documented in 55 countries with an average yearly incidence of approximately ten-thousand cases [2]. Infections occur through consumption of raw or inadequately processed meat or meat products containing the parasitic larvae [3]. Pork represents the most important source of human infection but meat of horses, wild boars, bears and badgers can also play a significant role [4]. Trichinellosis is mainly controlled through the examination of each animal carcass which may be infested with *Trichinella* and intended for human consumption [5]. Several countries have monitoring programs for assessing the prevalence of infection in wildlife and to identify the circulating etiological agents. According to the International Commission on Trichinellosis all positive samples should be forwarded to a qualified reference laboratory for the determination of the *Trichinella* species involved [6].

Currently, nine species, namely *T*. *spiralis*, *T*. *nativa*, *T*. *britovi*, *T*. *pseudospiralis*, *T*. *murrelli*, *T*. *nelsoni*, *T*. *papuae*, *T*. *zimbabwensis*, *T*. *patagoniensis* and three genotypes, *Trichinella* T6, *Trichinella* T8 and *Trichinella* T9, are recognized. [7, 8]. All species and genotypes can infect mammals. *T*. *pseudospiralis* also develops in birds and *T*. *papuae* and *T*. *zimbabwensis* can infect some reptile species [2]. *T*. *spiralis* and *T*. *britovi* are the most widespread etiological agents of *Trichinella* infection in wild and domestic animals and cause the most human infections worldwide. To date, the differentiation of the larvae to the species and genotype level is based primarily on molecular methods. Eight species and one genotype can be distinguished by multiplex PCR analysis [9, 10]. The remaining species and genotypes are identified by PCR-RFLP or PCR amplification and sequencing [7]. However, these methods are relatively time consuming.

In recent years, matrix assisted laser desorption / ionization time of flight mass spectrometry (MALDI-TOF MS) has evolved to a routine method for bacterial species identification due to its comparability to molecular methods, rapidness, minimum sample preparation and cost effectiveness per sample [11]. MALDI-TOF MS species identification is based on the protein profiling in which a unique spectrum is generated using the intact proteins extracted from an organism and compared with the spectra from the known organisms stored in the database through a pattern matching algorithm. Recently, we demonstrated that MALDI Biotyper, a commercial software, can also be used for the creation of reference spectra for species other than bacteria [12, 13]. As increasing number of routine diagnostics laboratories are implementing MALDI as a method of rapid microbial species identification, development of a *Trichinella* spectra database would be of great benefit without any extra cost or labor training. Therefore, the aim of this study was to establish a protocol for extraction of proteins and to create *Trichinella* specific reference spectra database using MALDI Biotyper software for rapid species identification similar to that of the microorganisms.

## Materials and Methods

### Sample collection

As listed in Table 1, nine *Trichinella* species and three *Trichinella* genotypes were utilized to establish a MALDI-TOF MS spectra reference library. All *Trichinella* isolates were passaged in mice and provided by Dr. Edoardo Pozio from the International *Trichinella* Reference Center. Larvae from domestic pigs, wild boar and guinea pigs originated from our own laboratory.

**Table 1:** List of *Trichinella* species and genotypes included in the study.

	*Trichinella* species/ genotype	host	ISS No
**T1**	*T*. *spiralis*	mouse	ISS 003, ISS 328, ISS 559
		guinea pig	ISS 003, ISS 1604
		domestic pig	ISS 003
**T2**	*T*. *nativa*	mouse	ISS 010, ISS 070, ISS 1606
		mouse	ISS 324, ISS 384, ISS 392
**T3**	*T*. *britovi*	guinea pig	ISS 002
		wild boar	ISS 5217
		mouse	ISS 176, ISS 4134, ISS 4556
**T4**	*T*. *pseudospiralis*	mouse	ISS 176, ISS 4134, ISS 4556
		guinea pig	ISS 013
**T5**	*T*. *murrelli*	mouse	ISS 035*, ISS 246*, ISS 346
**T6**	genotype T6	mouse	ISS 034, ISS 339
**T7**	*T*. *nelsoni*	mouse	ISS 037
**T8**	genotype T8	mouse	ISS 272*
**T9**	genotype T9	mouse	ISS 409
**T10**	*T*. *papuae*	mouse	ISS 572
**T11**	*T*. *zimbabwensis*	mouse	ISS 1029*
**T12**	*T*. *patagoniensis*	mouse	ISS 2311

*isolated from two individuals

*Trichinella* larvae were isolated from muscles using the magnetic stirrer method [14] and stored in ethanol (95%) at -18°C.

*Hyostrongylus rubidus* and *Trichuris* sp., obtained from domestic pigs, were utilized to assess the spectral specificity and were provided by Dr. Peter-Henning Clausen, FU-Berlin. Additional evaluation was also carried out using *Metastrongylus* sp., isolated from wild boar muscles and subsequently identified by 18S sequencing in our laboratory [15].

### Animal work and ethics statement

All animal work was conducted according to the guidelines of the German Protection of Animals Act (Tierschutzgesetz in der Fassung der Bekanntmachung vom 18. Mai 2006 (BGBl. I S. 1206, 1313). The State Office of Health and Social Affairs Berlin approved the described animal work (approval No. H 0078/00).

Two female guinea pigs (inbred strain 2A) were kept together in a 4800 cm^2^ large cage with 600g Licnocel Flakes and autoclaved hay (110g), with day light and a 12h light regime. There was a second floor in the cage with houses. The atmosphere was 20–22°C at 55% humidity. Water and food (‘Alleinfuttermittel für Meerschweinchen‘ by SNIFF) was given ad libitum. Animals were infected with 500 *Trichinella* larvae through non-invasive application by pipette. In total 4 guinea pigs were infected.

Two female domestic pigs (Deutsche Landrasse) were kept in a 9m^2^ box from 12 weeks of age. They were kept on approximately one bale of hay, had access to toys, a 12h light regime and atmospheric conditions as described for the guinea pigs. Water was freely available and the pigs were fed 1.2–2.5 kg/d pig feed from Ruckdeschel. One pig was infected with 40000 *Trichinella* spp. at 18 weeks through handfeeding of spiked food.

Both domestic pigs and small animals do not show any clinical signs of a *Trichinella* infection and did not need any treatment. Domestic pigs were electrically stunned inducing instantaneous insensibility, followed by bleeding. Guinea pigs were anesthetized using 0.4 mg ketamin and 0.1 mg xylazin/ kg body weight through intra-muscular application. At the time point of deep anesthesia, euthanization was performed by over dosage of CO_2_.

### Molecular species confirmation

The *Trichinella* species was confirmed by molecular techniques, which were defined as the gold standard. DNA extraction and PCR was performed as described by Pozio et al. [10] and Mayer-Scholl et al [16].

### Sample preparation for MALDI-TOF mass spectrometry

For the generation of the master spectra library (MSP) containing most reproducible peaks of the species /genotypes, a total of ten *Trichinella* larvae of same species or genotype were pooled and the protein extraction was carried out with 10 mg zirconia/silica beads (0.5 μm) and 100 μl 70% formic acid in a 2 ml Eppendorf tube. The samples were sonicated on ice for 3 min (amplitude 100%, pulse 60: UP200St, Hielscher Ultrasound Technology, Teltow, Germany). 100 μl 100% of acetonitrile were added and sonicated again. The suspension was centrifuged at 14.000 g for 3 min at room temperature and 150 μl of clear supernatant was collected for protein enrichment by vacuum centrifugation (12 min, 45°C, Eppendorf Concentrator 5301). One μl of each sample was spotted onto the target plate nine times (MSP 96 target polished steel (MicroScout Target) plate; Bruker Daltonik, Bremen, Germany), air dried and then overlaid with 1 μl of saturated α-cyano-4-hydroxy-cinnamic acid matrix solution (200 mg in 2.5% TFA/50% ACN) and dried completely.

### Influence of conservation methods, *Trichinella* hosts and number of larvae

The influence of different conservation methods such as freezing (*T*. *spiralis* infested meat at -20°C for 7 days) and 70% alcohol on the quality of the obtained spectra was measured. The influence of selected host species on the MALDI-TOF MS spectra mouse, guinea pig, domestic pig was also appraised. To determine if the method has a comparable sensitivity to the PCR, proteins were extracted from a single *T*. *spiralis* larva (fresh and frozen) and analyzed. All examinations were performed in triplicate. Further, to test the reproducibility of the method, *Trichinella* isolates IS 328, 559 and 003 were analyzed on different days by different technicians.

### Generation of *Trichinella* master spectrum library

The MALDI-TOF MS measurements were carried out using MALDI-TOF Microflex LT (Bruker Daltonics, Bremen, Germany) on a range of 2,000–20,000 m/z (mass to charge ratio) following the calibration with Bacterial Test Standard (Bruker Daltonics, Bremen, Germany). A total of 27 spectra per specimen were acquired from nine spots where each spot was measured three times. For each spectrum, 200 laser shots in 40 shot steps from different positions of the target spot (random walk movement) were automatically acquired using AutoXecute acquisition mode in Flex control 3.4 software. The quality of each spectrum was assessed with FlexAnalysis 3.4 software, the flat-liners and spectra with peak deviations exceeding 500 ppm were replaced with fresh measurements.

The composite correlation index (CCI) matrix was calculated with MALDI Biotyper 3.14 software with the following settings: lower bound 3,000, the upper bound 15,000, the resolution of the mass range four, and the number of intervals for CCI four. Following which, a master spectra library (MSP) was created using the default MSP creation method of the Biotyper software. The created master spectra were then compiled as a *Trichinella* specific MALDI-TOF MS spectra database. Master spectrum dendrogram cluster analysis was carried out with the correlation distance measure and single linkage. The threshold distance value for a single organism and zero for related organisms was computed based on the score value for species differentiation.

### Evaluation of generated spectra by score values

Score values function as a measure of the reliability of the isolate identification; these are acquired through the matching of the unknown spectrum against the reference library and vice versa and through the correlation of the intensities of the matched peaks. The following cutoff values recommended by the manufacturer were used for sample identification: 0 to 1.699 indicates ‘no reliable identification’; 1.7 to 1.999 indicates ‘probable genus identification’; 2.0 to 2.299 indicates ‘secure genus identification and probable species identification’, and 2.300 to 3.000 indicates a ‘highly probable species identification’.

## Results

**Fig 1:**
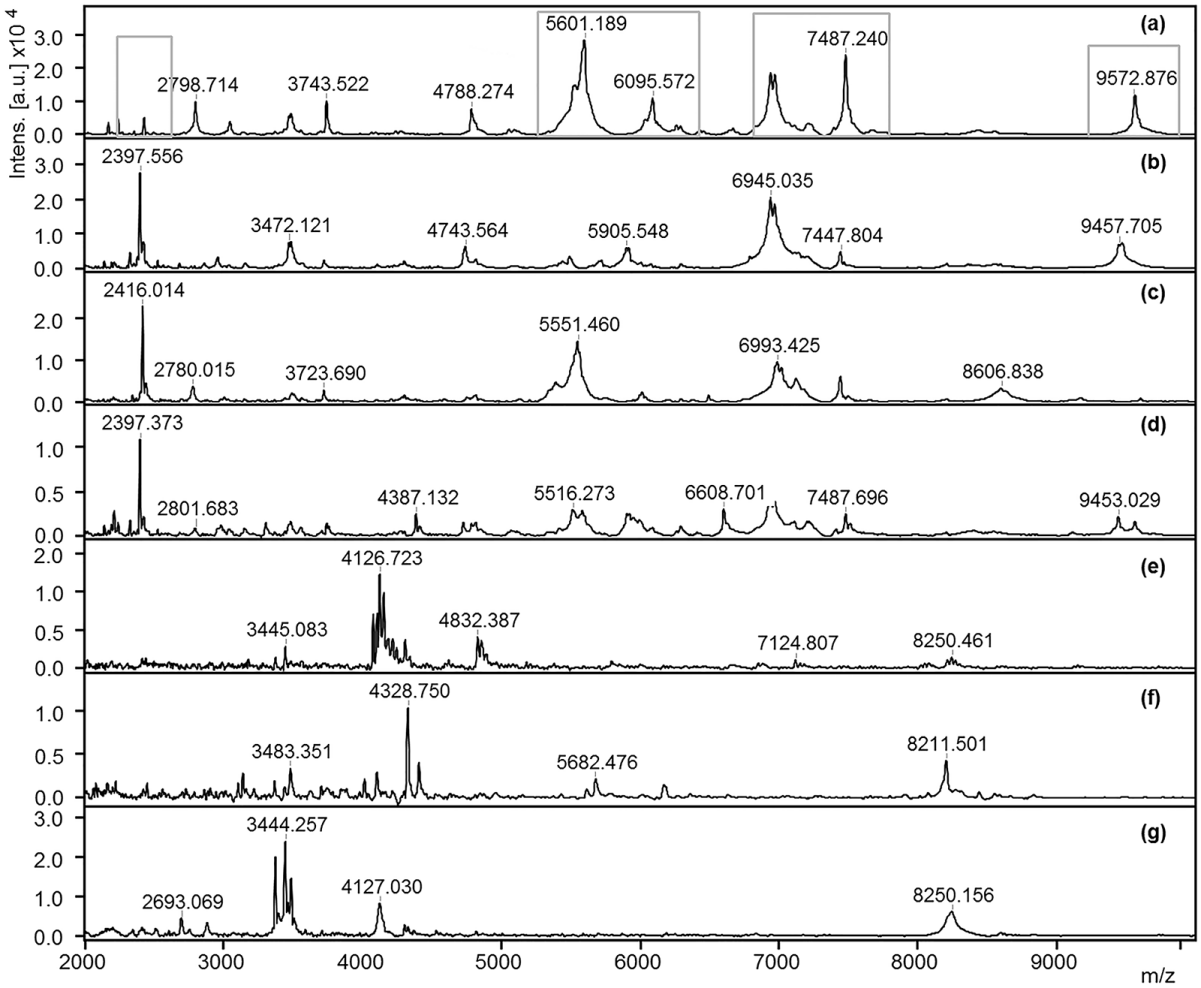
MALDI-TOF MS master spectra. *T*. *spiralis* (a), *T*. *britovi* (b), *T*. *pseudospiralis* (c) and *T*. *nativa* (d), *Trichuris* sp. (e), *Hyostrongylus rubidus* (f) and *Metastrongylus* sp. (g) on a zoomed range of 2,000–10,000 m/z (mass to charge ratio). Highlighted peaks are mere examples of visual difference observed between the isolates, e.g. through presence/absence of a peak or variations in terms of peak intensities.

The four *Trichinella* species (*T*. *spiralis*, *T*. *britovi*, *T*. *pseudospiralis* and *T*. *nativa*), which play the most important role in world-wide human infection clearly displayed different peaks, at least in terms of peak patterns and their intensities which indicates that the spectra are suitable for the creation of distinct reference spectra for each species (Fig 1). The nematodes included in the study to verify the specificity of the method displayed a different peak pattern in comparison to all *Trichinella* species (Fig 1, S1 and S2 Figs).

**Fig 2:**
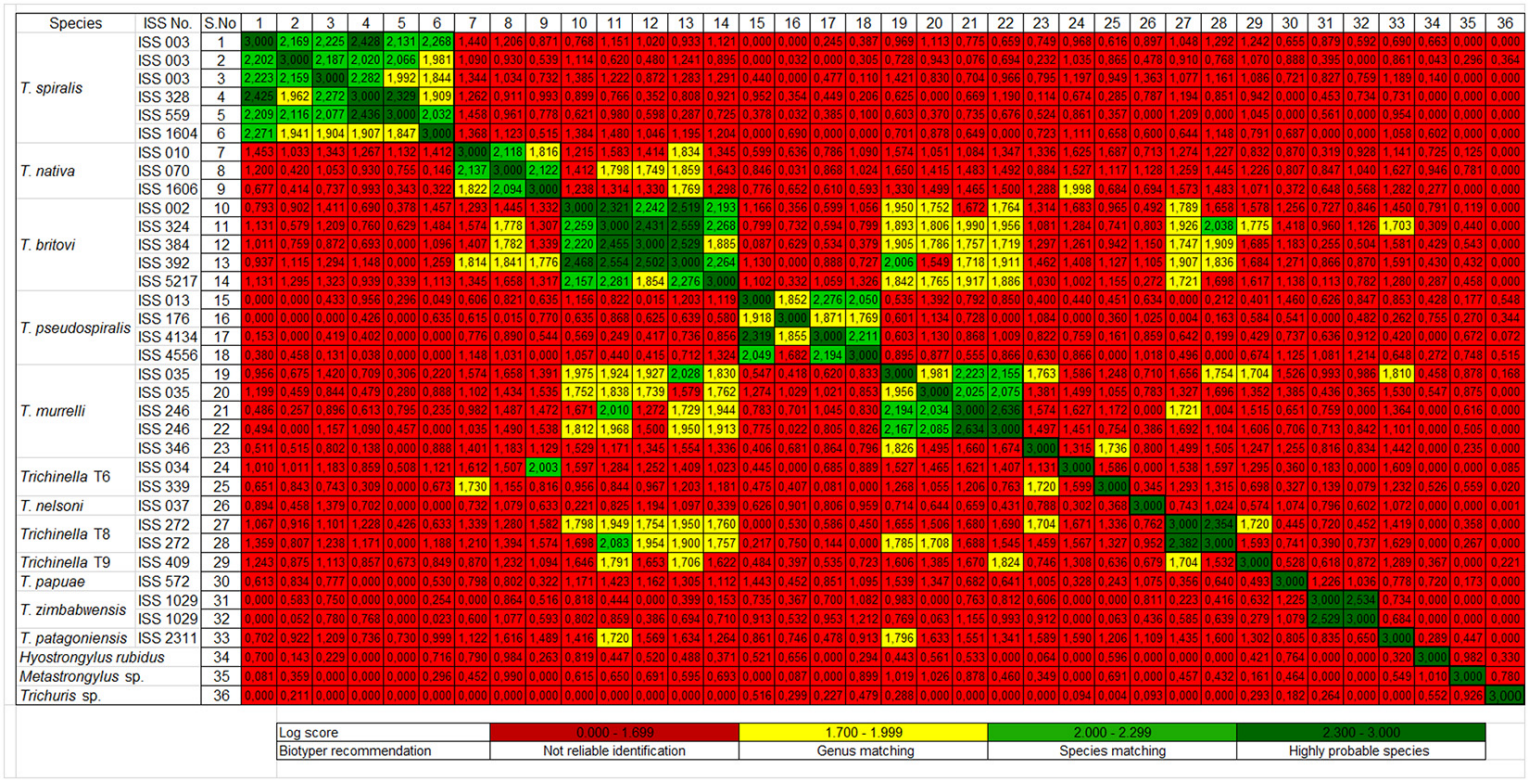
Heat map of all 33 tested *Trichinella* and 3 non-*Trichinella* strains based on log score values.

As shown in a heat map of all 33 tested *Trichinella* strains (Fig 2), all tested isolates of *T*. *spiralis*, *T*. *pseudospiralis*, *T*. *nelsoni*, *T*. *papuae* and *T*. *zimbabwensis* could be distinguished with high confidence. Some isolates within the *T*. *spiralis* species showed log score values between 1.7 and 1.99 (e.g. *T*. *spiralis* ISS 1604), however, interspecies comparison showed log score values lower than 1.70. *T*. *patagoniensis* and T9 could also be identified at the species level with high confidence, but congruencies on the genus level with other *Trichinella* species were identified (log score < 1.82).

The least reliable species classification could be seen for *T*. *britovi*, *T*. *nativa*, *T*. *murrelli*, T6 and T8. For example, the *T*. *murrelli* isolate ISS 346 only showed a ‘non reliable identification’ score value (1.50–1.83) when compared to three other *T*. *murrelli* strains. Further, two *T*. *murrelli* strains (ISS 035, ISS 246) showed a probable species identification with two *T*. *britovi* strains (ISS 392, ISS 324).

*Hyostrongylus rubidus*, *Metastrongylus* sp. and *Trichuris* sp. obtained log score values were lower than 1.10 (Fig 2). Comparison of the *Trichinella* spectra to the entire Bruker reference library did not reveal a single clear match. The highest log score value of 1.471 was found with *Lactobacillus crispatus*.

**Fig 3:**
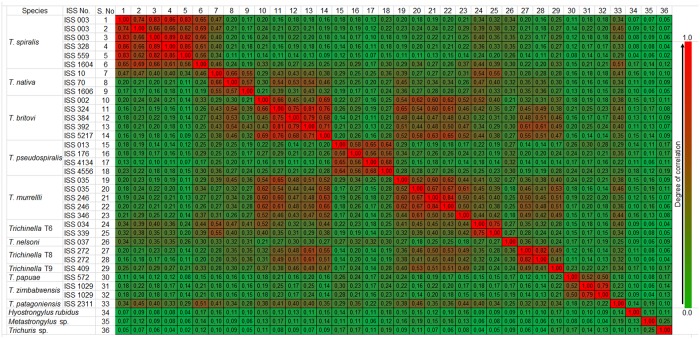
Composite correlation index analysis. Changes in color from red to blue correspond to decreasing degrees of correlation.

The relatedness of the spectra sets was analysed by composite correlation index analysis (Fig 3). The CCI ranged from 0.56 and 0.89 within the *T*. *spiralis* population, indicating high similarity. The highest inter-species CCI value for T1 was 0.51 with T12. The *T*. *pseudospiralis* isolates showed a lower degree of intra-species relatedness (CCI: 0.56–0.68), but a higher degree of incongruence with other *Trichinella* species (0.12–0.35). Conversely, *T*. *britovi* showed a high variability within the species (CCI: 0.39–0.81) but the highest degree of similarity with other *Trichinella* species, especially *T*. *murelli* (CCI: 0.39–0.65) and T8 (CCI: 0.31–0.61).

**Fig 4:**
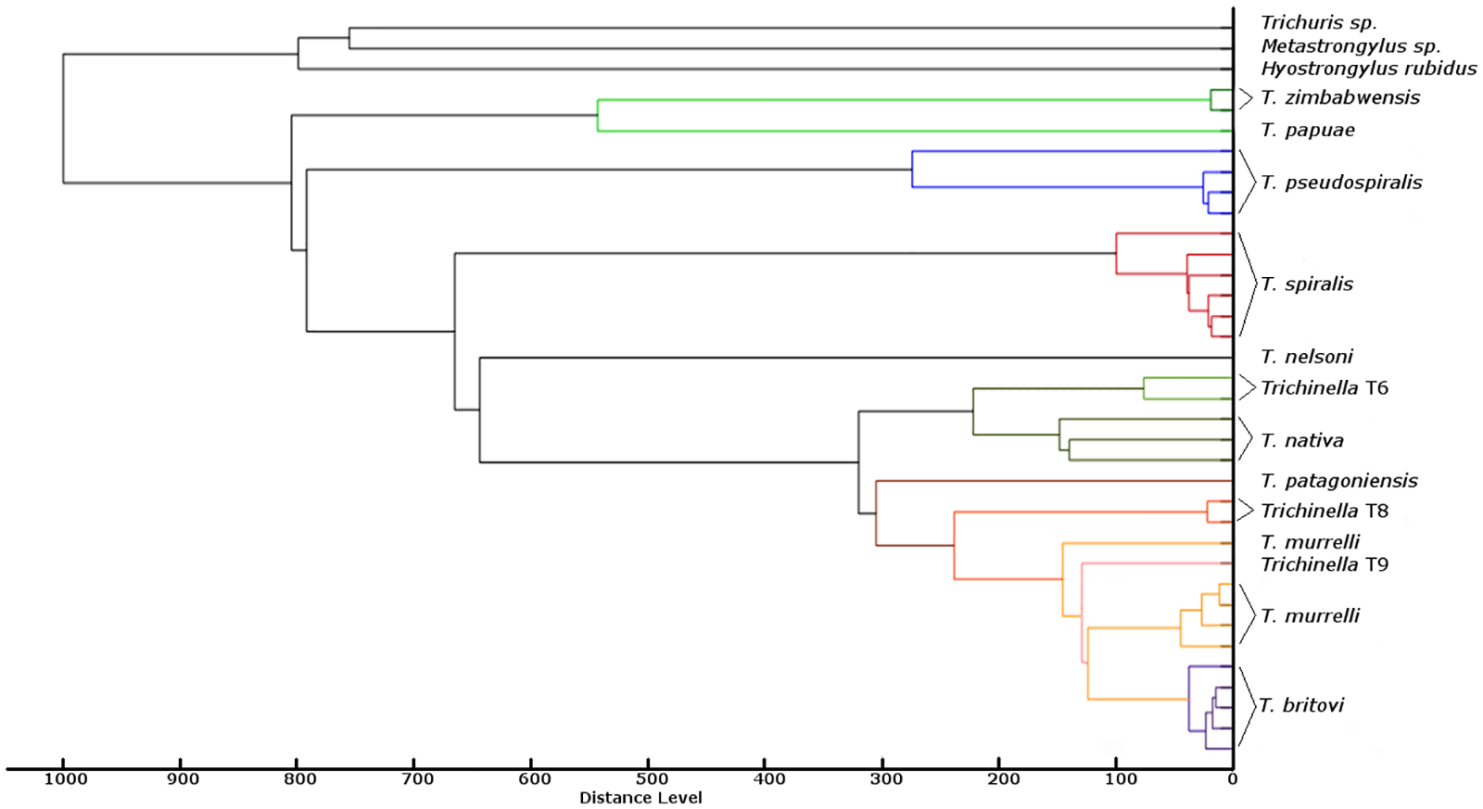
Master spectrum dendrogram cluster analysis (correlation distance measure and single linkage) for the *Trichinella* strains generated by MALDI-TOF MS.

As shown in Fig 4, the parasitic species *Trichuris* sp., *Metastrongylus* sp. and *Hyostrongylus rubidus* could be clearly separated from the *Trichinella* spp. cluster. All nine *Trichinella* species and three genotypes were separated into their distinct clusters, albeit with different levels of similarity. Only isolate ISS 346 (*T*. *murrelli*) did not form a cluster with the remaining four *T*. *murrelli* isolates.

Important criteria that could have an effect on the quality of the protein spectra and are important for future potential use in a routine setting include the reproducibility of the method, the larval host species, conservation methods, the number of larvae and the complexity of the protein extraction protocol.

The isolates tested on different days and by different technicians resulted in species level pattern matching log score values as recommended by the manufacturer: ISS 328 (2.5–2.6), ISS 559 (2.2–2.4) and ISS 003 (2.3–2.4), indicating good reproducibility and possible usefulness of this method for rapid species identification. Comparable peak patterns were found in ISS 003 larvae isolated from the three different host species (S3 Fig). The score values ranged between 2.16–2.23, which indicates probable species identification.

When generating MSPs using 10 larvae conservation methods (70% alcohol, freezing) did not have an effect on the log score values (2.3–2.6) or the peak patterns, in comparison to the log score values of native larvae (2.4–2.6)(). Only differences in peak intensities were identified (e.g. an intensity of 30000 vs 5000 in the major peaks). Also, fresh single larvae tested in triplicate did not show varying log score values (2.1–2.2) and could be identified with high confidence. In contrast, when larvae were frozen, quality protein spectra resulting in comparable log score values could only be generated using ≥5 larvae.

Only protein extraction protocols including sonication with beads resulted in high quality spectra (S4 Fig). As seen in S5 Fig, sonication with solvent and beads is necessary to completely disrupt the larvae to obtain sufficient proteins to correctly identify all *Trichinella* species.

## Discussion

In this study, we created a MALDI-TOF MS reference spectra database using *Trichinella* reference strains of all known species and genotypes. To date, two distinct *Trichinella* clades have been characterized by the presence or absence of an intramuscular collagen capsule [17]. *Trichinella* species that encapsulate in the host muscle tissue are *T*. *spiralis*, *T*. *nativa*, *T*. *britovi*, *T*. *murelli*, *T*. *nelsoni*, *T*. *patagoniensis* and the genotypes T6, T8 and T9. The second clade represents the *Trichinella* species which do not induce the formation of a capsule. Here, the species infecting mammals and birds (*T*. *pseudospiralis*) is clearly partitioned from those infecting mammals and reptiles (*T*. *papuae*, *T*. *zimbabwensis*) [7, 17]. As shown in the dendrogram, the evaluation of the spectra generated by MALDI-TOF MS mirrored the classification obtained by molecular methods.

As the current focus of our work was not the development of a taxonomic classification but to determine if the described method can be used in a routine setting, the discussion of the usefulness of the method will focus on the uniqueness of the created reference spectra.

The MALDI Biotyper and its integrated database were chosen to create a *Trichinella*-specific spectra database.

The four most prevalent species, *T*. *spiralis*, *T*. *britovi*, *T*. *pseudospiralis* and *T*. *nativa* resulted as highly probable species level matching with the correct species. Except in a few isolated cases, genus level matching was not observed among the strains belonging to the different species.

In contrast, two of the five *T*. *britovi* strains included in this study appeared to overlap with at least one strain of *T*. *murelli* and T8 at species level (>2.0). As *T*. *murelli* is the vicariant species of *T*. *britovi* in North America [7] difficulties in a reliable distinction between the two species is expected. Similar observations were also made among *T*. *nativa* and *T britovi* at genus level matching. However, this is not relevant as these stains matched with other strains of the same species with much higher log score values. This underscores that the database needs further improvement by addition of isolates representing several geographical regions and different hosts. Another possibility could be to define reduced log score cutoff values for species or genus level identification [18].

In routine *Trichinella* testing, fresh larvae are isolated from meat, identified morphologically and subjected to multiplex PCR analysis using one larva. Correct species identification was also achieved by MALDI-TOF MS when a single fresh larva was used. The larvae frozen at -20°C displayed reduced peak intensities, and therefore, 3–5 larvae are needed to obtain reliable spectra, which is comparable to the number of frozen larvae needed for the multiplex PCR.

Mixed *Trichinella* infections involving two separate species have been detected [19–21]. Through the visualization of additional bands, these infections can be detected by multiplex PCR, if more than one larva is examined. We found that samples containing two different *Trichinella* species are not identified reliably. In order to identify mixed infections by MALDI-TOF MS, a number of single *Trichinella* larvae need to be examined separately.

The mass spectrometry described here allows a fast verification of *Trichinella* larval isolates on genus level, which might be crucial for confirmatory testing after questionable findings during meat inspection. Furthermore, reliable determination of the four most prevalent *Trichinella* species worldwide is possible by means of MALDI-TOF MS analysis. As the method is easy to handle and can be performed within 1 hour, it represents a major step forward in the use of this technique in foodborne parasitology. Future studies will focus on the expansion of the *Trichinella* database to increase the accuracy and precision of identification and the validation of MALDI-TOF MS for typing of *Trichinella* field isolates. However, one should be cautious while interpreting the identification results up to the species and genotype level as this is the first attempt in a *Trichinella* database creation with a relatively very small number of isolates per species. It is proposed to reinforce the database with addition of strains representing various hosts and geographical regions. Furthermore, the database and protocol will be made available to large laboratories to test the performance of this technique under routine conditions.

## Supporting Information

S1 Fig MALDI-TOF MS master spectra of *Trichinella* spp. on a range of 2,000–20,000 m/z (mass to charge ratio). (TIFF)

S2 Fig MALDI-TOF MS master spectra of *Trichinella* spp. on a range of 2,000–20,000 m/z (mass to charge ratio). (TIFF)

S3 Fig MALDI-TOF MS master spectra of *Trichinella spiralis* ISS 003 larvae isolated from three different host species. (TIFF)

S4 Fig MALDI-TOF MS master spectra of *Trichinella spiralis* larvae where protein extraction was performed a) without sonication and b) with sonication. (TIF)

S5 Fig Effects of different protein extraction protocols on larval integrity. (A) *T*. *spiralis* larvae in water. (B) *T*. *spiralis* larvae sonicated w/o beads in 70% formic acid and 100% acetonitrile. (C) *T*. *spiralis* larvae sonicated with beads in 70% formic acid and 100% acetonitrile.
(TIF)
