# Supplementary figures and images for: Rapid Identification of the Foodborne Pathogen Trichinella spp. by Matrix-Assisted Laser Desorption/Ionization Mass Spectrometry
Source: PLoS One. 2016 Mar 21;11(3):e0152062. doi: 10.1371/journal.pone.0152062 (PMC4801418; doi:10.1371/journal.pone.0152062)

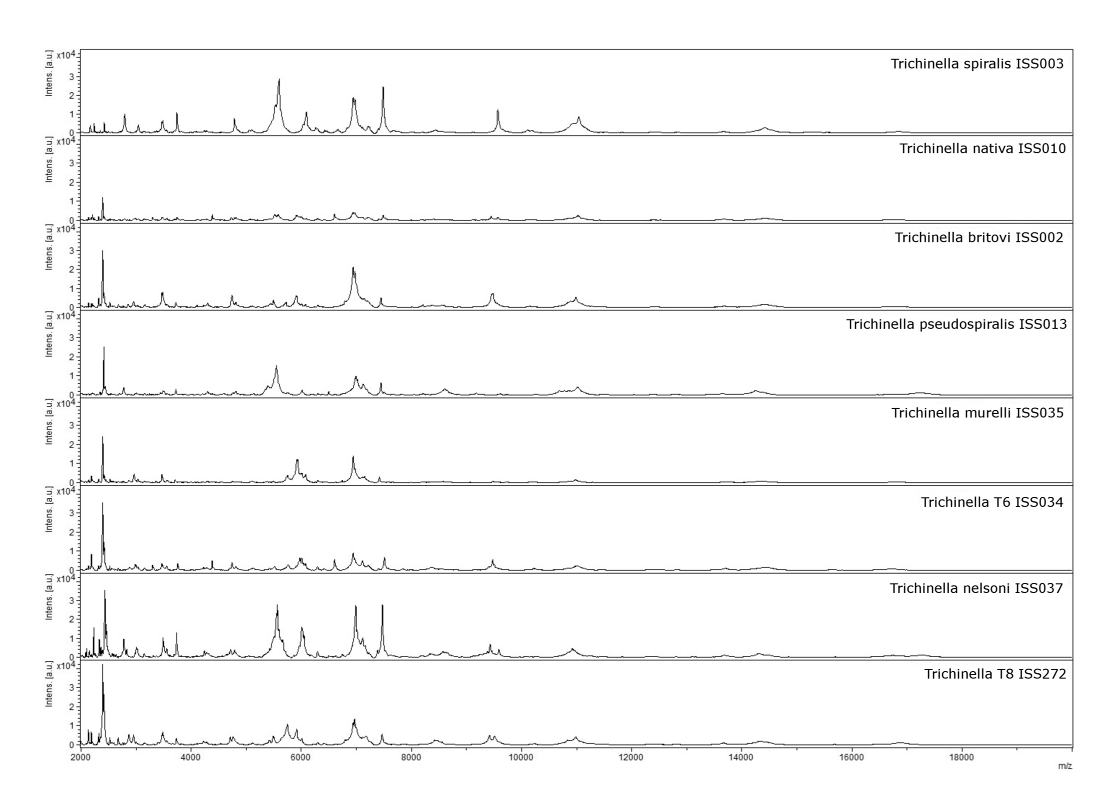

Supplement: S1 Fig — (TIFF) [file pone.0152062.s001.tiff]

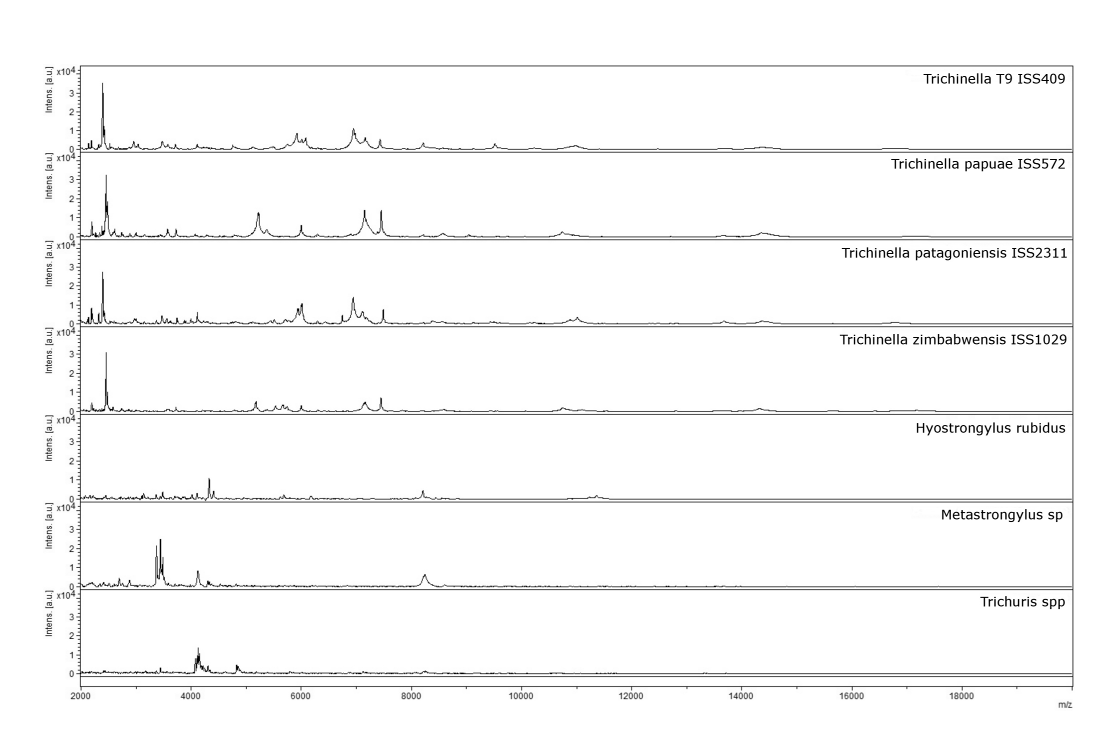

Supplement: S2 Fig — (TIFF) [file pone.0152062.s002.tiff]

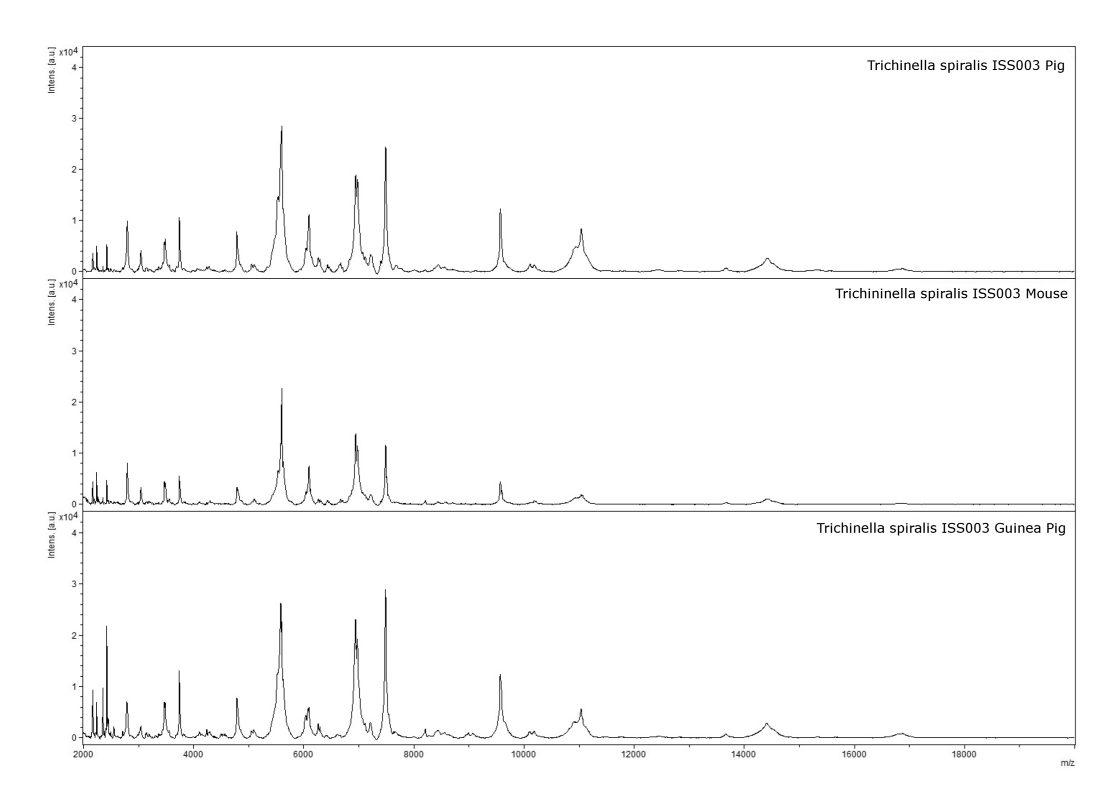

Supplement: S3 Fig — (TIFF) [file pone.0152062.s003.tiff]

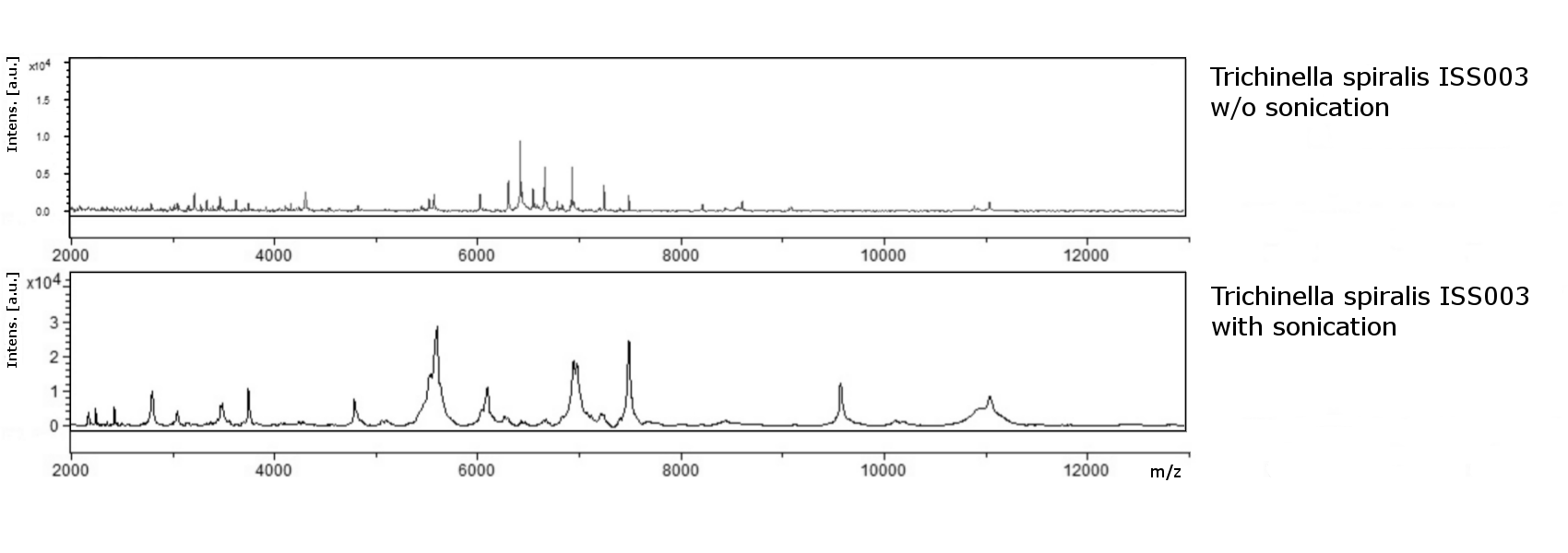

Supplement: S4 Fig — (TIF) [file pone.0152062.s004.tif]

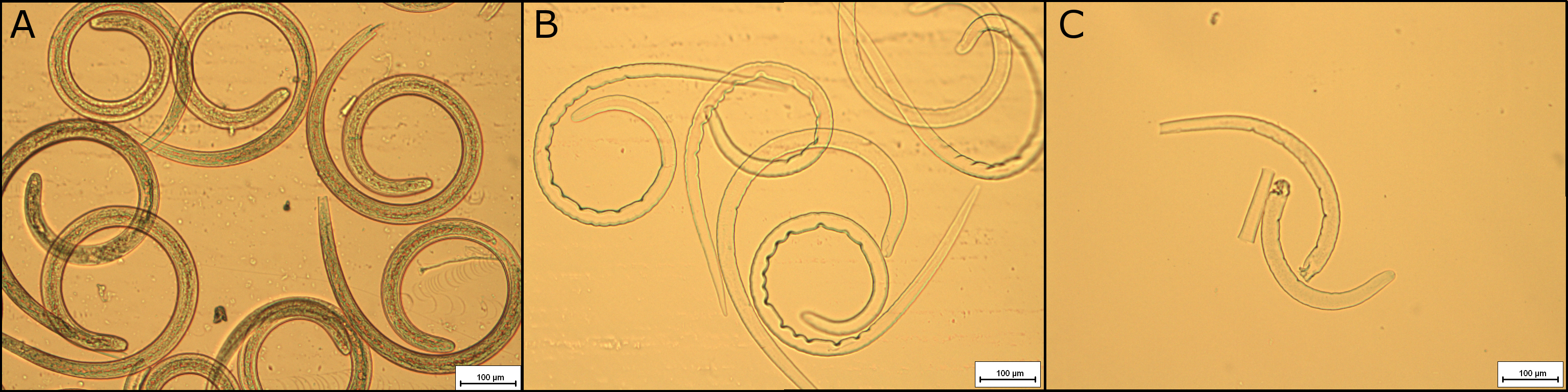

Supplement: S5 Fig — (A) T. spiralis larvae in water. (B) T. spiralis larvae sonicated w/o beads in 70% formic acid and 100% acetonitrile. (C) T. spiralis larvae sonicated with beads in 70% formic acid and 100% acetonitrile. (TIF) [file pone.0152062.s005.tif]
